# Reshaping the peripersonal space in virtual reality

**DOI:** 10.1038/s41598-024-52383-y

**Published:** 2024-01-29

**Authors:** Irene Petrizzo, Kyriaki Mikellidou, Savvas Avraam, Marios Avraamides, Roberto Arrighi

**Affiliations:** 1 Department of Neuroscience, Psychology, Pharmacology, and Child Health, University of Florence, 50135 Florence, Italy; 2 Department of Management, CIIM Business School, University of Limassol, Limassol, Cyprus; 3 Department of Psychology & Center for Applied Neuroscience, University of Cyprus, Nicosia, Cyprus; 4 CYENS Centre of Excellence, Nicosia, Cyprus

**Keywords:** Perception, Sensory processing, Object vision, Human behaviour

## Abstract

Peripersonal space (PPS) is defined as the space that lies within reach. Previous research revealed that PPS can be dynamically reshaped with the use of tools extending the arm’s reach. Here we investigated whether PPS reshaping depends on the kind of selected tool and/or the motor routine associated with its use. Participants carried out a visuo-tactile detection task in an immersive VR environment that allowed to measure the PPS size before and after a short period of tools use. In Experiment 1, participants had to pull or push objects towards or away from themselves using a shovel. In Experiment 2, they were required to either hammer or shoot an avatar placed in the Extrapersonal space. We found, for the first time in a VR environment, that a period of *pull* training was effective in enlarging the PPS, a result that replicates and expands previous findings carried out in real life conditions. However, no significant change in PPS size was achieved for training with other tools and motor routines. Our results suggest that the reshaping of PPS is a complex phenomenon in which the kind of interaction between the agent, the targets and the exploited motor routines all play a critical role.

## Introduction

The space around us can be divided based on what lies within our reach or outside of it. This distinction is of primary importance for defining efficient motor plans that allow us to interact with the environment^1^. The area surrounding our body, which is known as the *Peripersonal space* (PPS), was first described in monkeys, with the discovery in the ventral periarcuate cortex of multi-modal neurons that only fire if a stimulus is placed near the body of the animal^2^. Interestingly, the electrical stimulation of these neurons elicits defensive-like movements in monkeys, as if the animals are trying to protect the part of the body corresponding to the receptive fields of the stimulated neurons^3^. This evidence seems consistent with the idea that the PPS might be conceptualized as a system specifically dedicated to the perception of stimuli that are in the immediate surroundings of the body that can be reached with the hands and may be of potential risk or interest^4^. In line with that, it has been proposed that the activity of brain areas in which PPS neurons are located aims at maintaining a margin of safety around the body^5^.

Neuropsychological studies with neglect patients provide evidence for the presence of a specific area in the human brain dedicated to the perception of stimuli in the PPS. Hemineglect, or simply neglect, is a condition resulting from brain damage that leads to the inability to attend to stimuli presented in the contralesional hemifield^6^. In some cases, neglect patients fail to report the presence of a stimulus presented in the contralesional side when a competing stimulus is simultaneously shown in the ipsilesional side, a phenomenon known as “extinction”^7^. Ladavas et al.^8^ demonstrated that the extinction also occurs cross-modally: the reduction in sensitivity to a tactile stimulus triggered on the contralesional hand induced by an ipsilesional touch was rather identical to that yielded by a visual stimulus displayed around the ipsilesional hand, suggesting the existence of a cross-modal visuo-tactile extinction. However, this effect was significantly reduced when the visual stimulus was presented outside of the patient’s PPS, indicating that the deficit in the combination of visual and tactile information in neglect was more pronounced within the PPS^8^. A dissociation between PPS and the Extrapersonal space (EPS) was also found in pseudo-neglect, an attentional deficit presented by neurologically healthy individuals. When asked to bisect a horizontal line, most people tend to provide leftward-biased responses^9^. However, this attentional bias attenuates progressively with increasing distance: when asked to perform the bisection task in EPS, individual responses shift rightward instead of leftward^10,11^.

Much evidence suggests that the PPS is distorted in clinical populations with both congenital^12,13^ or acquired disorders^14,15^, but changes in its size have also been reported in the typical population as a consequence of long term conditioning. Indeed, as the size of the PPS is robustly based on the extension of our reach, if our reach changes, the PPS changes accordingly. Specifically, when monkeys are trained to use a stick to retrieve food from a distance, the receptive fields of PPS neurons become longer^16^. Similar evidence has also been collected in humans, with reports of amputees^17^ and wheelchair users (considering the PPS around the feet)^18^ having a smaller PPS compared to controls, whereas blind cane users^19^ and long-term computer mouse users^20^ have a larger PPS when holding their cane or mouse respectively. Also, elite athletes show signatures of PPS remapping. For instance, experienced tennis players exhibit larger PPS when they hold their racket^21^, with a similar effect also being found in fencers holding their sword^22^.

While long-term conditioning has been reported to induce a stable and durable reshaping of the PPS, transient changes in PPS can also be achieved as a consequence of short training sessions. For instance, after performing a task in which participants use the tip of a cane to find objects scattered on the floor, an extension of the PPS was reported^19^. This effect was similar to blind cane-users, however the remodulation in the healthy participants was transient: when participants were retested the day after the training, the PPS extension had disappeared^19^. This result supports the idea that the duration of the training is proportional to the stability of the remapping. Indeed, this brief extension of PPS as a result of a short tool-use training has been observed under several different conditions^17,20,23–25^. An enlargement of the PPS has also been observed while walking on a treadmill^26^, suggesting that the illusion of moving forward, even when there is no overall displacement, increases the perception of what can be considered within reach. Finally, the size of the PPS can also be modulated according to the social validity of the stimuli in the surrounding space. Geers and Coello^27^ have recently found that when a human avatar was present, even if it was not interacting with the participant, their extent of reachable-space was significantly reduced.

Despite much evidence suggesting that the expansion of PPS is achievable in many different ways, for example via the execution of specific motor routines, it is not clear which aspect of the training triggers the reshaping. Is it the forward motion of a limb, the proprioceptive feedback of reaching with a tool, or the forward motion of the whole body? While the general definition of the size of the PPS is straightforward, its precise measurement poses some challenges. In animal models, the border between PPS and EPS can be measured with accuracy using in-vivo single-cell recording^2^. However, in humans a non-invasive behavioral approach needs to be adopted. One of the most widely used methods to capture the boundary between PPS and EPS is the audio-tactile detection task pioneered by Canzonieri et al.^28^. In this task, participants were presented with a looming auditory stimulus that creates the illusion of an approaching sound source. Following a predetermined delay, a tactile stimulus is delivered to the hand as a vibration and participants are instructed to react to this tactile stimulus as fast as possible, while ignoring the auditory stimulus. If the vibration is delivered when the sound is perceived as being in the PPS, participants are faster to react than when the sound is perceived as being in the EPS. Thus, the operational definition of PPS using this auditory-tactile detection task can be summarized as the maximum distance from the participant’s body at which the auditory stimulus can still facilitate the detection of the tactile stimulus. Notably, one of the characteristics of PPS neurons is that they are multimodal^2^ and thus capable of firing for both visual and auditory impulses. This has opened to the possibility of measuring detection facilitation within the PPS with visual instead of auditory stimuli, but the design of reliable approaching visual stimuli posits some technical challenges. Virtual Reality (VR) set-ups offer an ideal environment to understand which aspects of the training plays a key role in inducing the reshaping of the PPS as it allows to design ecologically plausible looming visual stimuli. Indeed, previous reports suggest that the PPS can be successfully investigated with VR^27,29–31^. As it has been demonstrated for the audio-tactile task^28^, if the vibration is delivered when the virtual visual stimulus is perceived as being close to the participants body, reaction times (RTs) to the vibration are significantly reduced, and in some cases almost halved^29^.

In our study we wanted to leverage on a VR environment to study the malleability of PPS size by attempting to induce a PPS reshape with four different kinds of tool-use training. In Experiment 1, tool training consisted of participants using a shovel to move objects placed on the floor away from their body, towards them. In a different condition, participants were instead required to do the opposite movement: to push objects placed near them, further away into the far space. While in both experimental conditions, the motor routine of the hand was identical, the visuo-motor interaction between the participant and the object was not. In one case (Push) the object was initially located near the body of the participant and then moved away. In the other condition (Pull) the object was initially located far from the participant’s body and then moved near them. In case the key factor driving the resizing of the PPS was the motor routine executed with the hand, we expect to observe identical results in the Push and Pull conditions. On the contrary, if the key factor for PPS reshaping was the *direction* the objects were moved to during the training, the Push and Pull conditions will have different effects on the size of the PPS.

In Experiment 2 we aimed at investigating whether additional kinds of motor training, not requiring the movement of objects from the near to far space or vice versa (as in Experiment 1), can reshape the PPS. In one condition, we asked participants to repeatedly hit a target located in the EPS using a hammer with a long handle, while in another condition the direct physical interaction between the operator and the targets was minimized by asking them to aim and shoot the target with a toy-gun.

In general, if the PPS in VR retained the same properties as in real-world settings, we expected to replicate the finding of the Pull training as an effective motor routine to induce a PPS resizing as observed in real-life settings. However, no a-priori hypotheses were instead posited for the other three conditions as the essential characteristics for PPS resizing by using tools has never been investigated before.

## Results

### Experiment 1

#### Data preprocessing

**Figure 1:**
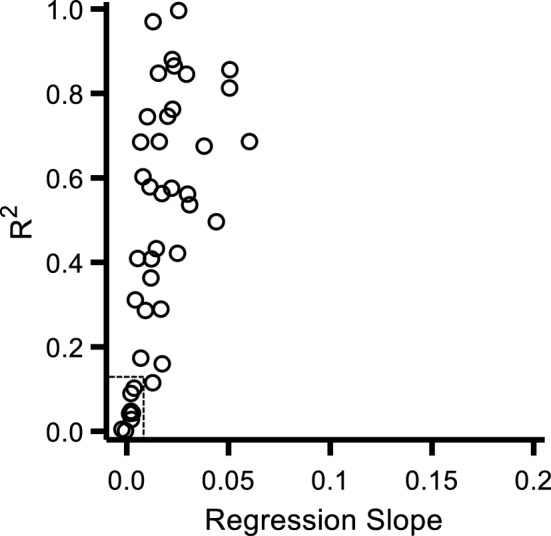
Correlation between R^2^ and the slope of the linear fit for each participant’s RTs in the baseline condition of Experiment 1. Individual average RT as a function of visual stimulus distance were fit with a linear regression model. As suggested by previous literature, RTs for vibration detection were expected to increase as a function distance. The goodness of fit and the slope predicted by the model for each participant were then correlated with each other to reveal a strong positive correlation. Participants with the lowest goodness of fit values and flattest slope (shown within the dotted rectangle at the bottom left of the graph) were excluded from further analyses, bringing down the sample size from 41 to 33.

At the beginning of each experiment, we employed the visuo-tactile detection task to measure the size of the PPS for each participant (baseline condition). On each trial a tactile vibration was delivered through the hand controller and participants were instructed to press a button as soon as they detected it. At the same time an irrelevant visual stimulus was delivered, namely a bubble that travelled mid-air at eye level towards participants who were instructed to ignore it. The vibration was delivered when the bubble was at one of five possible distances away from the participant. Participants’ reaction times were used as an index to determine the size of the PPS. As there is an extensive literature showing that during a visuo-tactile or audio-tactile detection task participants RTs decrease as the irrelevant stimulus approaches^17,26,29–31^, we looked for an increase in RTs in the baseline condition as a function of visual stimulus distance at which a tactile vibration was delivered. For each participant, we measured the linear regression (Eq. 1) and then correlated the variance explained by the linear model (R^2^) with the slope of the fit (*b* in Eq. 1). As expected, we found a strong correlation between the two (Pearson’s r = 0.61, p < 0.001). Data in Fig. 1 show that participants with the steepest slope, indicating a steeper increase in RTs as a function of visual stimulus distance, also had the best (i.e. highest) goodness of fit to their data. Furthermore, there is a clear cluster of participants who had both a very poor goodness of fit (R^2^ < 0.1) and a very flat slope (*b* < 0.005), suggesting that not only RTs did not increase as a function of stimulus distance but also did not follow a linear trend in general (bottom left in Fig. 1, datapoints within dotted rectangle). In other words, these participants showed no reduction in RTs as the irrelevant stimulus was approaching, but the variance of the whole dataset was poorly accounted for by a linear model indicating poor performance in general. For this reason, this cluster of 8 participants was excluded, leaving data from 33 participants (out of 41) to be used for further analyses.

#### Size of PPS before and after training

**Figure 2:**
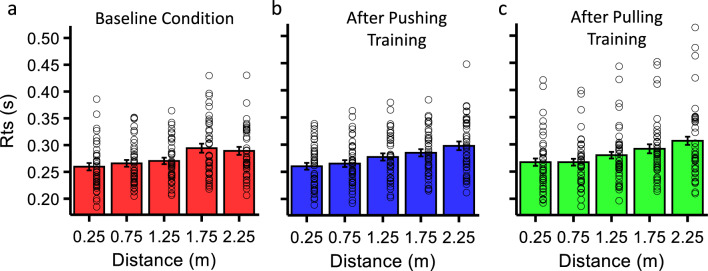
RTs as a function of visual stimulus distance in Experiment 1. RTs for the baseline condition (in red) are shown in the leftmost panel while data for the two training conditions, Push and Pull, are shown in the middle and right panel respectively. Hollow circles represent individual RTs, vertical bars depict average RTs ± 1 S.E.M. In all conditions RTs increased as the distance of the irrelevant visual stimulus from the participant at the time the tactile vibration was delivered, increased.

Individual and average RTs for each irrelevant visual stimulus distance are plotted in Fig. 2 for the baseline and the two experimental conditions in which the PPS was measured after the tool use training. In all three conditions, there was a clear influence of stimulus distance on RTs, with longer distances triggering the slowest RTs which ranged between 250 and 310 ms (averaged across participants) and are in line with previous reports^31^.

To test whether the PPS of participants had changed as a result of the training performed in the Push and Pull conditions, individual RTs for the trials in which the ball was launched from the central monster (80% of all trials) were pooled together and analyzed at the group level. In the Pull training participants had to use a shovel to pull objects scattered on the floor towards them, while in the Push training they had to use the same tool to push objects away from them. To make the three conditions easier to compare with each other, average RTs were normalized between 0–1 using Eq. (2). To determine the exact border of the PPS in the three conditions average RTs were plotted as a function of visual stimulus distance and fitted with a sigmoid function (Eq. 3). The point of maximum slope of the fit (x_c_) was considered as the border of the PPS^31^.

**Figure 3:**
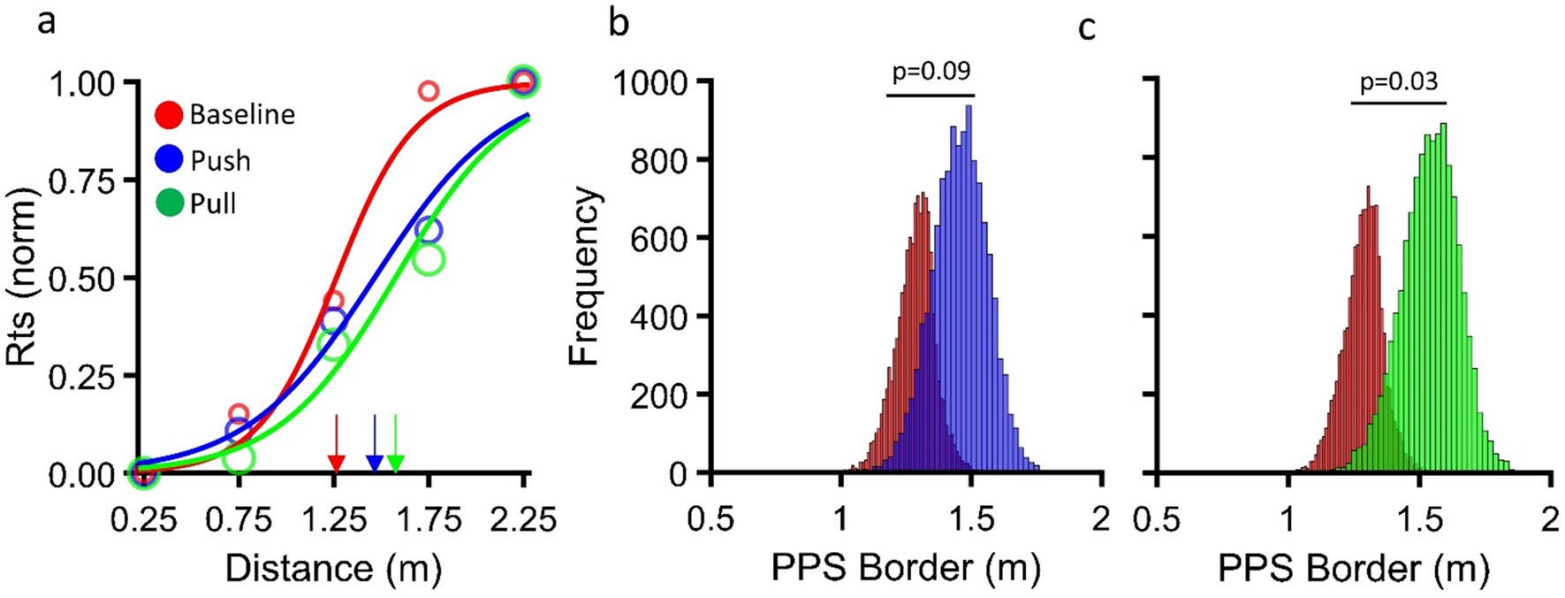
RTs sigmoid fit and bootstrapping. (**a**) Average RTs (normalized between 0–1 range) plotted against visual stimulus distance and fitted with a sigmoid function. The point of maximum slope of the fit (x_c_) was considered the border between the PPS/EPS. Each curve indicates the size of the PPS before and after a given type of training. (**b**) Bootstrap distributions for the baseline (red) and Push (blue) conditions. Each column represents how many times a certain value of PPS size was obtained out of 12,000 bootstrap iterations. The difference between the two distributions is then evaluated with a bootstrap t-test. (**c**) Bootstrap distributions for the baseline (red) and Pull (green) conditions. In this case, the difference between the two distributions was statistically significant.

Aggregate sigmoid functions for the three separate conditions are plotted together in Fig. 3A. The PPS appears to be the smallest in the baseline Condition (red curve) with an average size of 1.26 m, while the PPS appears to get enlarged as a result of both the Pull (green curve) and the Push training (blue curve), yielding a PPS measuring 1.58 m and 1.46 m respectively. The size of the PPS in the baseline condition is almost identical (only a difference of 1 cm) to the one previously found by Buck et al.^29^ using the same paradigm in VR.

The significant difference between the aggregate data for each condition was quantified by the bootstrap sign test. On each one of the 12,000 iterations, separately for each condition, the data were sampled with replacement (to yield as many independent samples as the full dataset) and fitted with a sigmoid function, to estimate the border of the PPS. For the Push condition (Fig. 3B) there was a slight trend of PPS enlargement after training, as shown by the peaks of the distribution that were rather spread apart. However, this trend did not reach statistical significance (p = 0.09). For the Pull condition (Fig. 3C), the same trend of a PPS enlargement after training was observed but this time modulation was quantitatively higher and statistically significant (p = 0.03). These results suggest that, in line with several previous reports, the Pull Training yielded a significant enlargement of the PPS of around 20% (0.32 m of difference between the two conditions). The Push training, despite including a motor routine in the opposite direction (from PPS to EPS), also showed a tendency to induce an enlargement of the PPS, however its failure to reach statistical significance suggests that the direction of motion routines is important in inducing a reshaping of the PPS.

### Experiment 2

#### Data preprocessing

**Figure 4:**
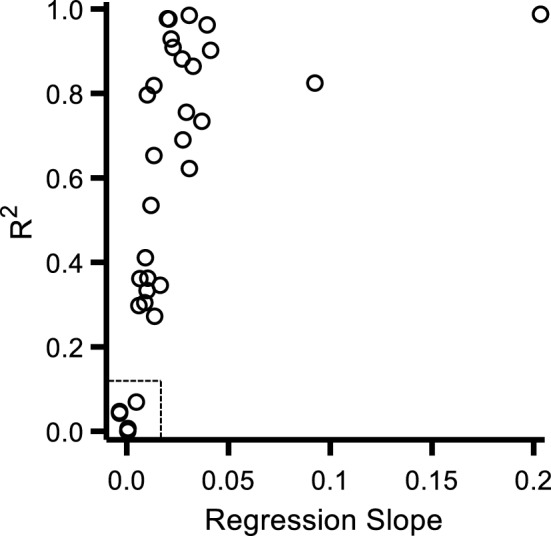
Correlation between R^2^ and the slope of the linear fit for participants’ RTs in the baseline condition of Experiment 2. Individual average RTs as a function of irrelevant visual stimulus distance were fitted with a linear regression model. The goodness of fit and the slope predicted by the model for each participant were then correlated with each other. Participants with the lowest goodness of fitting values and flattest slope (within the dotted rectangle, bottom left) were excluded from further analyses.

In the second experiment, we tested the role of training motor routines that (a) did not involve crossing the PPS/EPS border or (b) did not include a direct, physical contact between the observer and the target in EPS. As a preliminary step, and similarly to Experiment 1, individual RTs were fitted with a linear function to confirm a linear increase as a function of visual stimulus distance. Also in this case, we found a strong correlation between the variance explained by the linear model (R^2^) and the Regression Slope (*b*, in Eq. 1). The two values were strongly correlated with each other (Pearson’s r = 0.49, p = 0.003) and again we found a small cluster of participants (within the dotted rectangle of Fig. 4) who exhibited both a very low goodness of fit and a very flat slope. These participants that provided noisy and stereotypical responses were excluded from subsequent analyses, resulting in a total of 27 participants in the final dataset (total tested: 32).

#### Size of PPS before and after training

**Figure 5:**
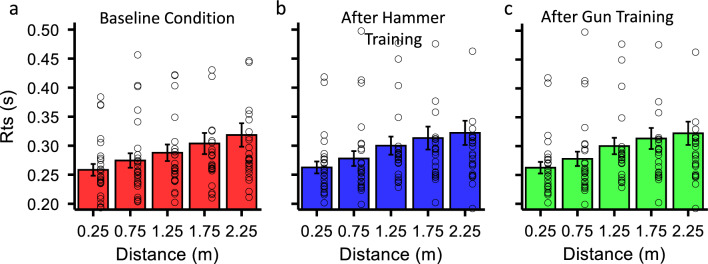
RTs as a function of visual stimulus in Experiment 2. RTs for all experimental conditions (baseline, Hammer and Gun conditions) to detect the tactile vibration for all distances between the approaching visual stimulus and the observer. Hollow circles represent individual RTs, vertical bars depict average RTs ± 1 S.E.M. In all three conditions RTs to detect the vibration increased as the distance between the observer and the approaching visual stimulus increased.

Average RTs in the three conditions are plotted as a function of approaching visual stimulus distance in Fig. 5. Experiment 2 confirms the validity of the visuo-tactile detection task as a tool to measure the border of PPS, given that again, RTs steadily increase as the distance of the irrelevant visual stimulus at which a tactile vibration is delivered increases. Furthermore, average RTs are in line with Experiment 1 (250 to 310 ms), ranging from 250 to 320 ms.

To test whether the Hammer and Toy-Gun training had any influence on the size of the PPS, all trials in which the ball was launched from the central monster (80% of all trials) were pooled together and analyzed at a group level. First, average RTs were normalized between 0 and 1 using Eq. (2). Then, average RTs as a function of visual stimulus distance were fitted according to Eq. (3) (see Methods) and the point of maximum slope (x_c_) was taken as the border of PPS.

**Figure 6:**
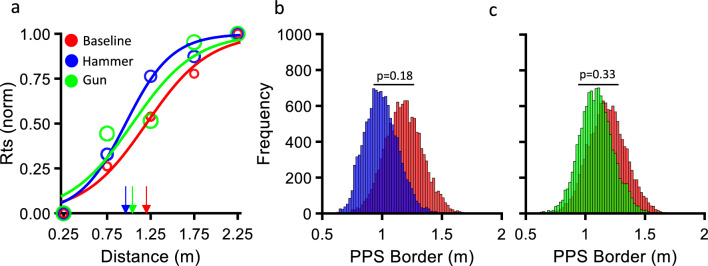
RTs sigmoid fit and bootstrapping for Experiment 2. (**a**) Normalized average RTs plotted against the irrelevant visual stimulus distance. The data distribution was fitted with a sigmoid function and the point of maximum slope of the fit (x_c_) defined as the border of PPS. Each curve indicates the size of the PPS before and after each type of training. (**b**) Bootstrap distributions of the PPS size for the Baseline and Hammer conditions. Each column represents how many times a certain PPS size was obtained out of 12 000 iterations. The difference between the 2 distributions is then tested with a bootstrap t-test. (**c**) Bootstrap distributions comparing the baseline and Toy-Gun conditions. None of the two experimental conditions yielded significantly different results from the baseline condition.

Sigmoid functions on aggregate data across participants for the three separate conditions are plotted in Fig. 6A. The size of the PPS was the biggest in the baseline Condition (red curve-1.20 m). This result is very close to the size of PPS found in the baseline Condition of Experiment 1 (i.e., 1.26 m). Indeed, there was no statistically significant difference between the baseline conditions of two experiments (p = 0.27), as tested with the same bootstrapping procedure implemented in Experiment 1.

At odds with Experiment 1, the two trainings employed in Experiment 2 did not induce any significant changes of the size of PPS. Both conditions yielded a slight shrinkage of the PPS, that was more pronounced in the Hammer condition (0.96 m) than in the Gun Condition (1.04 m) and in contrast with the PPS enlargement reported for the Pull training of Experiment 1. However, neither the use of the hammer (Fig. 6B) nor that of the Toy-Gun induced a significant change on the size of the PPS (Fig. 6C). The results of the two experiments combined suggest that (i) objects we interact with should be crossing the PPS/EPS border and (ii) direct physical contact between the tool and the object of interest are two essential factors for PPS resizing.

## Discussion

In this study we investigated the effects of a brief training period of tool-use on the size of the PPS in a VR setting. Across two experiments, participants had their PPS measured before and after performing four different types of tool-training. We measured the size of PPS by leveraging on a visuo-tactile detection task, which has been shown to be a valid method for determining the size of the PPS in humans^31^. In the baseline (pre-training) condition of both experiments we found that the extension of PPS was 1.26 m and 1.20 m respectively. This result validates the visuo-tactile paradigm in VR as it is almost identical with previous findings^29,30^.

In Experiment 1, the size of the PPS was measured after two kinds of training: the Pull training in which participants had to pull marbles near them from the afar and the Push training in which participants had to push marbles away from themselves and into the far space. We found that the Pull training triggered a significant enlargement of the PPS of more than 20% after only 10 min of training. This result is in line with previous reports that used a similar training motor routine in animal models^16^, brain-damaged patients^32–34^, and healthy controls^17,35^. However, this is the first time in which an expansion of the PPS is achieved in VR via tool-use training, thus providing key evidence for the effectiveness of using VR environments to measure and modulate the PPS. Even after the Push training, in which the interaction with the objects was carried out in the opposite direction (from near to far space), we found a trend for an expansion of the PPS (around 15%) but this effect was quantitatively less robust and failed to reach statistical significance. To further investigate how different motor routines during training can modulate the PPS size, in Experiment 2 we designed two new training conditions. In the first, participants had to use a hammer to hit a blob avatar that was moving sideways in the EPS, while in the second condition they had to use a toy-gun to shoot the avatar moving along the same semi-circular trajectory. Although we did observe a slight tendency towards a shrinkage of the PPS, none of the two types of training had any significant effect on the size of the PPS.

Considering the results of Experiment 1, one might speculate that the direction of motion during the training is crucial, with the Pull routine requiring to move objects from the EPS to the PPS being the only kind of training capable of remapping PPS. Indeed, there have been previous reports of action specificity on the extension of the PPS suggesting that different action kinematics might yield different effects. For instance, Brozzoli et al.^36^ compared the effects of a grasping and a pointing action on visuo-tactile interactions. They found that during the approaching phase of the movement, the grasping action led to a stronger cross-modal congruency effect compared to the pointing action, in which participants approached the target without touching it.

The Hammer condition of Experiment 2 was devised to test whether a tool training paradigm in which there is no object moving from far to near distance (or vice versa), as it was the case in Experiment 1, was effective in distorting the size of PPS. However, our data have shown that this is not the case. Similarly, also a training with a tool that did not encompass any direct physical contact between the participant and the targets (Toy-Gun) failed to significantly affect the dimension of the PPS. However, the conditions exploited in Experiment 2 might also be seen from a different perspective. The Hammer and Toy-Gun training might be considered two kinds of fight-related actions allegedly capable of activating a defensive behavior: as participants were asked to use “weapons” to hit the blob avatar, this might have triggered the implicit belief of having to defend themselves from the avatar. Indeed, it has been established that carrying out a defensive behavior for a prolonged period can influence the PPS. For instance, expert boxers exhibit an anomalous Hand Blink Reflex (HBR) compared to controls^37^. The HBR is a subcortical response at the brainstem level, elicited by the electrical stimulation of the median nerve at the wrist and recorded from the orbicularis oculi muscles. HBR dramatically increases when the stimulated hand is statically positioned inside the PPS surrounding the face^38^. However, when boxers assume the guard position (with both hands protecting their face), the HBR is heavily suppressed, even though the affected limb is stationarily positioned within the PPS. This might be because boxers perceive themselves as protected from danger while they are in the guard position, thus shrinking their PPS. However, the hypothesis of Experiment 2 triggering a defensive behavior and thus preventing an expansion of the PPS as a consequence of the training is not supported by our results, as no significant effect was observed. Another possible explanation for the lack of significant results after the Hammer training is that the size of the tool (as the Hammer was longer than the Shovel and the Toy-gun) might have rescaled the visual field by changing the apparent distance of the elements in the scene, thus nulling any possible effect induced by the extension in reach provided by the Hammer. Even if this hypothesis was correct, it could not be generalized to account for the lack of significant effects reported after the Toy-Gun training to suggest that further experiments have to be designed better understanding the critical variable needed to distort the PPS size.

One of the key contributions of the present study is that it has successfully investigated PPS manipulations in a VR environment, as previous studies have questioned the possibility to do so. For instance, Ferroni et al.^39^ found that the same kind of training that triggered an expansion of PPS in real life did not yield the same result in VR, which prompted them to conclude that the PPS in VR might be characterized by different properties. One possible explanation to reconcile these findings might be in terms of the differences between the experimental paradigm implemented here and in Ferroni et al.^39^. In their paradigm, during the training phase participants had to move an object from point A to point B with both points located in the EPS, with no transitions between the near and the far space. This means that participants never had to move objects closer to or further away from them. On the other hand, in the Pull training of the present study, the only condition that was successful in inducing a significant enlargement of the PPS, objects were moved from EPS to PPS to establish a clear connection between the two spaces. Considering this, it is surprising that the opposite movement, Push, did not provide any significant result and even more interesting is the fact that, nevertheless, the tendency found was towards an enlargement of the PPS. If the direction of motion is crucial to the reshaping, one might have expected a constriction of the size of the PPS. Going back to the comparison between the present study with that of Ferroni et al.^39^, it is clear that the condition more similar across the two studies is the Hammer training, in which participants had “physical contact” with the targets but without moving the objects between the PPS and the EPS. Notably, in both Ferroni et al.^39^ and here such training yielded only a weak non-significant tendency to shrink the PPS. These results, in combination with those from the present study, strongly suggest that the type of motor routine carried out during the training, plays a fundamental role in PPS reshaping.

From the current results it is clear that pulling objects towards oneself has a special role in the representation of PPS, which could possibly be derived from the evolutionary relevance of pulling objects closer in order to interact with them. Future studies should aim at investigating two main experimental questions. First, it should be determined whether it is possible to trigger a reshaping with any other kind of tool training while considering variables such as the kinematics of the training actions or the training duration. Subsequently it would also be crucial to test the lifespan of the expansion effect triggered by the tool training, to determine whether changes in the PPS are transient phenomena or are long-lasting. Finally, as the present study revealed that reliable and accurate measurements of PPS can also be achieved in VR, future studies should take further advantage of this technology to investigate PPS in more ecological settings and scenarios.

## Methods

### Participants

A total of 46 participants took part in Experiment 1—Push & Pull (mean age: 21.43 ± 3.85 years old, 37 females, 1 author). 33 participants took part in Experiment 2—Hammer & Gun (mean age:22.12 ± 3.37 years old, 24 females, 1 author). Two participants took part in both experiments. All participants had normal or corrected-to-normal vision and gave written informed consent. The study was approved by the Cyprus National Bioethics Committee (Protocol Number: EEBK EP 2018.01.138) and was in accordance with the ethical standards of the 1964 Declaration of Helsinki. Participants were asked to abstain from caffeine for at least two hours prior to the experiment to control for possible effects induced by stimulants on RTs^40^. Due to technical failure of hardware, complete behavioral datasets from 6 participants were not possible to collect and were thus excluded from further analyses, leaving a total of 41 (out of 46) participants for Experiment 1 and 32 participants (out of 33) for Experiment 2.

### Apparatus

The VR environment was built in Unity Game Engine (version 2020.3.29f1) using C# version 7.3. The blob avatar and the tools animations were imported from the Unity Asset Store. The virtual environment was presented to participants using the HTC Vive ProEye headset, that features a resolution of 2880 by 1600 pixels and a nominal refresh rate of 90 Hz. All data was analyzed using Matlab 2020b (The Mathworks, Inc., Natick, MA, USA).

### Measurements of PPS: baseline task

**Figure 7:**
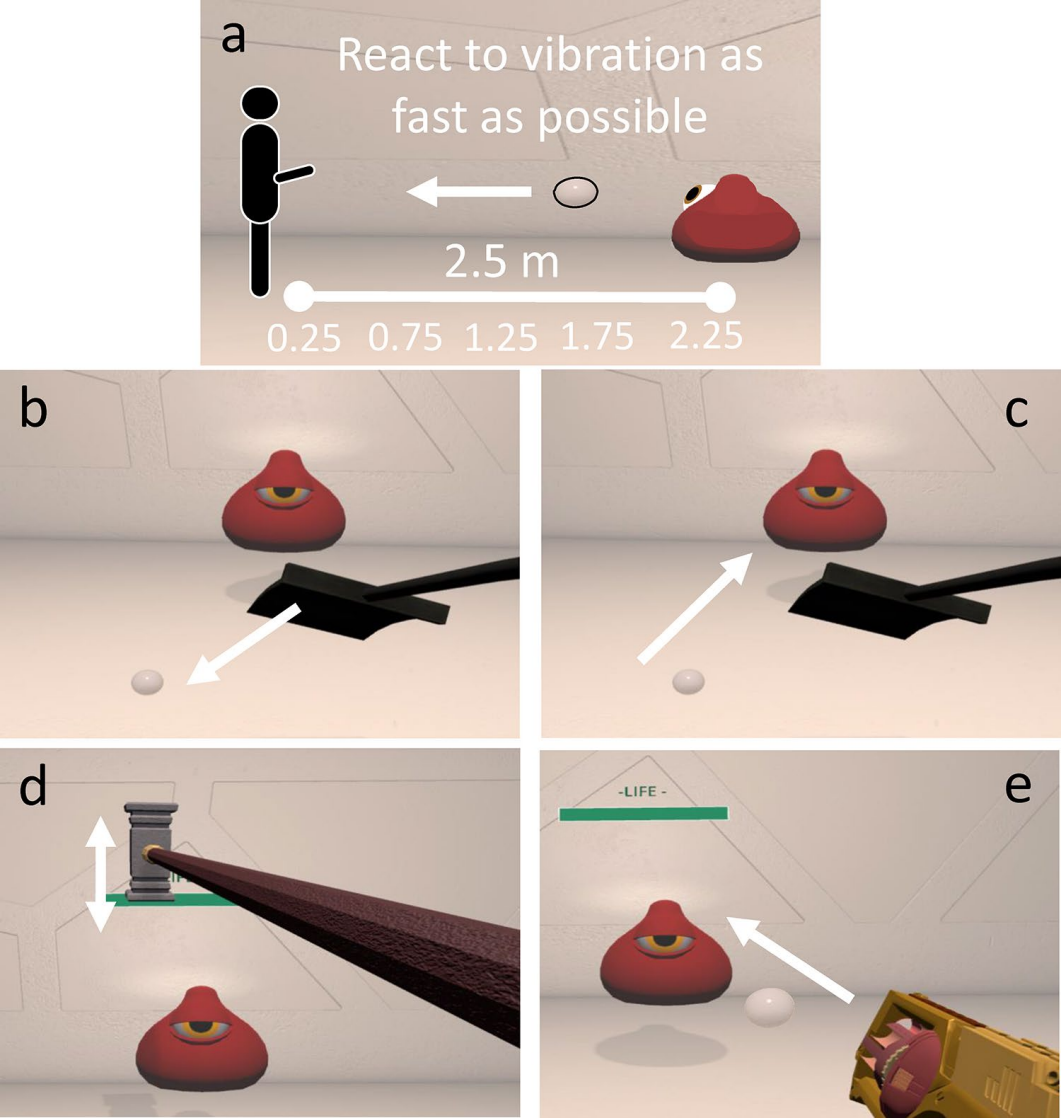
Experimental Paradigm. (**a**) Measuring of PPS. Participants were instructed to react to a vibration delivered through the hand controller as fast as possible, while ignoring an approaching visual stimulus. The vibration could be delivered when the visual stimulus was at 5 possible distances from the participant. The monster avatars were placed at 2.5 m of distance from the participant, and they bubble approached them at a constant speed of 0.75 m/s. (**b**) Push Training: On each trial a marble appeared on the floor and participants were instructed to use the shovel to push it towards the avatar. Once the marble had reached the inside of a circle with a radius of 0.7 m placed around the avatar, the marble disappeared, and a new trial started. (**c**) Pull Training: this was similar to the Push training but requiring an opposite direction of motor movement. Participants used the same shovel to pull the marble towards their feet. (**d**) Hammer Training: participants used a hammer to hit the avatar using an up-and-down vertical motion, while the avatar moved from left to right following a semi-circular trajectory of motion. A Green Life Bar decreased at each successful hit. (**e**) Toy-gun Training. Participants used a gun to shoot the avatar, the same Life Bar used in the Hammer Training was used to signal the progression of the task. The avatar kept moving from left to right on the same path of motion used in the Hammer condition.

To measure the size of the PPS we used the visuo-tactile detection task first proposed by Serino^31^. The experiment took place in a neutral virtual room in which three blob avatars were positioned at a distance of 2.5 m away from the observer, either straight ahead (at 0°) or at an eccentricity of 30° to the left or to the right of the observer (Fig. 7A). The avatars remained still for the whole duration of the task apart from an idle animation: slight up-and-down bouncing on the spot and blinking of their only eye. Participants were instructed to (i) stand still inside a small circle which ensured a fixed distance (2.5 m) from the blob avatars, (ii) fixate on the central blob avatar, and (iii) pull the trigger on the controller each time they felt a vibration. On each trial, one of the avatars (the central avatar on 80% of the trials) launched a semi-transparent bubble with a diameter of 10 cm that travelled horizontally at a constant speed of 75 cm/s towards the participant. The vibration through the hand controller could be delivered when the bubble was at 2.25, 1.75, 1.25, 0.75 or 0.25 m away from the participant. In total we collected 20 repetitions for each distance in Experiment 1 and 15 repetitions for each distance in Experiment 2 to optimize the duration of the Task. On top of that, 15 trials for each of the two control conditions were added: (1) tactile only, in which the vibration was delivered without a bubble being launched, and (2) visual only, in which the bubble travelled the whole distance with no vibration being delivered. The control conditions ensured that the appearance of the ball did not trigger a stereotypical response. This meant that on each PPS measurement the participant was presented with a total of 140 trials for Experiment 1 and 105 trials for Experiment 2, with a single block lasting approximately 10 min.

### Experiment 1—push and pull

#### Pull training

The participant was positioned in the same non-descriptive white room used for the baseline Task described above, with only one blob avatar placed at 2.5 m directly in front of them (Fig. 7B). The blob avatar did not move except for the idle animation. On each trial a small round marble appeared on the floor between the participant and the blob avatar at a maximum distance of 1.5 m from the participant and at an eccentricity of 0°, 30° or 60° on the left or the right respectively, at one of five possible positions. Participants were instructed to use a shovel (1.45 m long) to pull the marble into a circle of 70 cm radius, while standing in its center. This translated in each marble having to be moved for a total distance of 0.8 m to reach the target circle. Once the marble had been successfully dragged into the circle, the next trial was initiated. Each participant completed a total of 200 trials, with 40 trials for each possible eccentricity presented in a pseudorandom order. The whole block lasted approximately 10 min.

#### Push training

The methods were very similar to those for the Pull training, with the key difference being the direction of the motor routine to perform. Specifically, on each trial a marble appeared at a maximum distance of 1.5 m from the avatar eccentricity of 0°, 30° or 60° on the left or the right respectively of the avatar. This translated in each marble having to be moved for a total distance of 0.8 m to reach the target circle, the exact same travelling distance as the Pull condition. Participants had to use the same shovel used in the Pull Condition to push the marble away from them into a circle of 70 cm radius positioned around the blob avatar, instead of pulling it towards them as in the previous condition. This task used in Experiment 1 aimed at testing whether the direction of action while using a tool plays a role in reshaping the PPS (Fig. 7C).

### Experiment 2: hammer and toy-gun

#### Hammer training

In this condition participants were positioned in the same non-descriptive white room with a single blob avatar standing at fixed distance of 2.5 m and moving from left to right in a semi-circular trajectory reaching a maximum of 30° of eccentricity on each side at a speed of 2.618 m/s (Fig. 7D). Participants were equipped with a hammer bridging the distance between their extended arm and the blob avatar and were instructed to hit the avatar on the head. Each successful hit was signaled by both a vibration of the controller and a back-and-forth rocking motion of the avatar (as if it was reacting to the hit). Furthermore, to make the task more engaging, a green life-bar representing the remaining “energy” or “life” of the avatar was positioned above it. On each successful hit, the lifespan decreased, reaching 0 after 200 successful hits. Each participant performed three blocks of this training in succession, with a total duration of about 10 min (same as in previous trainings).

#### Toy-gun training

Participants had to use a hand controller which was animated as a yellow toy-gun in the virtual reality environment to hit the blob avatar that moved along the same trajectory described for the hammer training. Every time the trigger was pulled the controller vibrated and each successful hit was signaled by a back-and-forth movement of the blob avatar (similar to the hammer training). The bullet travelled towards the target at a speed of 2.618 m/s. The same life-bar used for the hammer training was used here as well to let the participant know how long the training would last for. Each participant completed 3 blocks of 200 trials. (Fig. 7E).

### Procedure

Independently of the experiment and the order of conditions, the experimental session began with presenting participants with a 7-trial sample of the baseline Task (one for each of the 5 possible distances over which the vibration would be delivered through the hand controller, 1 for the tactile only condition and 1 for the visual only condition) in order to familiarize them with the procedure.

Participants performed the baseline Task to measure the extension of their PPS three times in each experiment: once without having performed any training (baseline Condition) and once after each training session (Push Condition and Pull Condition for Experiment 1 or Hammer Condition and Toy-Gun Condition in Experiment 2). The order of conditions was counterbalanced across participants. Between each condition participants removed the VR headset and took a 15-min break to ensure that any remaining effect induced by training wore off before beginning the next condition of the experiment.

### Data analysis

To measure the size of the PPS we pooled together trials from all participants in which the bubble was released by the central monster (80% of all trials) and analyzed them at the group level. During the data preprocessing all trials in which participants failed to pull the trigger or pulled the trigger before the vibration, were excluded. To further ensure that we only considered genuine responses to the tactile stimulus, we also excluded trials in which the RTs were unreasonably fast (< 100 ms-possibly indicating a reaction to the release of the bubble and not the vibration) or slow (> 1000 ms-possibly indicating lack of attention) or fell beyond 3 standard deviations from the average individual RTs of each participant, for each possible distance. In practice, this led to the removal of less than 1% of all trials, which were excluded from further analyses and not replaced.

There have been various reports in the literature showing that RTs in a visuo-tactile or audio-tactile detection task are expected to increase as a function of increasing irrelevant stimulus distance^17,26,29–31^. For this reason, in a preliminary analysis we plotted individual RTs against visual stimulus distance and fitted them with the linear function (Eq. 1):

1 $$y\left(x\right)=ax+b$$

The computed *b* coefficient indicates the slope of the fitting function, with b coefficients closer to 0 indicating a lack of trend in RTs as a function of distance, which led to participant exclusion.

In order to make RTs easier to compare among conditions, average RTs at the group level were normalized between 0 and 1 (Eq. 2)

2 $${z}_{i}=\frac{\left({x}_{i}-min\left(x\right)\right)}{max\left(x\right)-min\left(x\right)}$$

To compute the exact PPS border in each condition, average RTs at the group level were plotted against visual stimulus distance and fitted with a sigmoid function (Eq. 3) similarly to Serino et al.^31^. Fitting parameters were set to anchor the sigmoid curve between 0 and 1, and x_c_ was restricted between 0.25 and 2.25 m (our shortest and longest distance over which vibration was delivered).

3 $$y(x)=\frac{{y}_{min}+{y}_{max}{e}^{(x-xc)/b}}{1+{e}^{(x-xc)/b}}$$

Statistical significance was tested using the bootstrapping method^41^. On each repetition (12,000 iterations) and separately for each condition, the data were sampled with replacement (as many independent samples as the full dataset i.e., a new dataset with the same size of the original was obtained on each repetition) and fit with the sigmoid function described above, whose peak yielded an estimate of the size of PPS. As an additional step to ensure that each tested distance was represented equally in the new re-sampled dataset, each tested distance was resampled an equal number of times, thus preventing a possible unbalanced dataset. For Experiment 1, each distance was resampled on average 532 times in each new dataset while in Experiment 2 each distance was resampled on average 323 times. PPS size distributions for each condition were tested for significance separately using a Bootstrap t-test^41^.

## Data Availability

Data for the main findings of this study are available at: 10.5281/zenodo.8072114.
